# Antimicrobial Peptide Induced-Stress Renders *Staphylococcus aureus* Susceptible to Toxic Nucleoside Analogs

**DOI:** 10.3389/fimmu.2020.01686

**Published:** 2020-09-29

**Authors:** Alexandro Rodríguez-Rojas, Arpita Nath, Baydaa El Shazely, Greta Santi, Joshua Jay Kim, Christoph Weise, Benno Kuropka, Jens Rolff

**Affiliations:** ^1^Institut für Biologie, Evolutionary Biology, Freie Universität Berlin, Berlin, Germany; ^2^Zoology Department, Faculty of Science, Alexandria University, Alexandria, Egypt; ^3^Institute of Chemistry and Biochemistry, Freie Universität Berlin, Berlin, Germany

**Keywords:** pexiganan, antibiotic resistance, antimetabolites, antimicrobial peptides, antibiotics, nuceloside analogs

## Abstract

Cationic antimicrobial peptides (AMPs) are active immune effectors of multicellular organisms and are also considered as new antimicrobial drug candidates. One of the problems encountered when developing AMPs as drugs is the difficulty of reaching sufficient killing concentrations under physiological conditions. Here, using pexiganan, a cationic peptide derived from a host defense peptide of the African clawed frog and the first AMP developed into an antibacterial drug, we studied whether sub-lethal effects of AMPs can be harnessed to devise treatment combinations. We studied the pexiganan stress response of *Staphylococcus aureus* at sub-lethal concentrations using quantitative proteomics. Several proteins involved in nucleotide metabolism were elevated, suggesting a metabolic demand. We then show that *Staphylococcus aureus* is highly susceptible to antimetabolite nucleoside analogs when exposed to pexiganan, even at sub-inhibitory concentrations. These findings could be used to enhance pexiganan potency while decreasing the risk of resistance emergence, and our findings can likely be extended to other antimicrobial peptides.

## Introduction

Antimicrobial peptides (AMPs, we use AMPs here as synonymous with host defense peptides) are immune effector molecules used by multicellular organisms to control infections (1–3). These peptides are usually active against a broad spectrum of bacterial pathogens and some display activity against antibiotic-resistant bacteria. Thus, antimicrobial peptides are considered a promising source of new antibacterial drugs (4, 5) to tackle the current antibiotic crisis (6).

Some of the factors that make AMPs attractive are their high diversity across the tree of life (7) and the finding that, although drug resistance also evolves against AMPs (8–12), it evolves at a much lower probability in comparison to conventional antibiotics (3, 13, 14). One common problem with the development of AMPs as drugs is that, under physiological conditions, their antimicrobial activity cannot be easily recaptured and the required dosage is extremely high (15). This dosage issue can be addressed by making use of synergistic combinations of AMPs (16), a property common in natural defense cocktails (17, 18).

While the mode of action on bacterial membranes has been worked out for some AMPs (19), the consequence of AMP-induced stress on bacterial physiology is less studied. The first goal of this study, therefore, is to understand how the pathogen *Staphylococcus aureus* reacts to different doses of pexiganan at the minimum inhibitory concentration (MIC). Pexiganan is a drug that was mostly developed against this bacterium (20). This molecule is a 22-amino-acid peptide (Gly-Ile-Gly-Lys-Phe-Leu-Lys-Lys-Ala-Lys-Lys-Phe-Gly-Lys-Ala-Phe-Val-Lys-Ile-Leu-Lys-Lys-NH2); molecular weight, 2478 Da [free peptide base] and has cationic nature. It is a derivative analog of the magainin II peptide isolated from the skin of the African clawed frog *Xenopus laevis*. Pexiganan exhibited broad-spectrum antibacterial activity *in vitro* when tested against 3,109 clinical isolates of gram-positive and gram-negative, anaerobic and aerobic bacteria (20). Pexiganan shows a barrel-stave type mechanism of membrane disruption (or channel formation). The consensus is that Pexiganan exerts its antibacterial effect by forming toroidal pores in the bacterial membranes. Pexiganan effectively induced the uptake and leakage of small compounds from both bacterial membranes and *in vitro* assembled lipid vesicles (21).

Using pexiganan as an example, we found that different concentrations induce the upregulation of several genes depending on nucleotides or related to nucleotide metabolism. Based on these results, we hypothesized that this would lead us to identify phenotypic collateral sensitivity. We hypothesized that the response to pexiganan sensitizes *S. aureus* against certain nucleoside antimetabolites or toxic nucleoside analogs. Interestingly, these analogs have been proposed as an alternative to antibiotics as a consequence of resistance emergence (22). Nucleoside analogs have the advantage of being clinically approved for cancer therapies, but also as antiviral and antifungal treatments (22). Pyrimidine and purine analogs, as we use here, showed potent antimicrobial activity against *S. aureus* in the past (22–25).

In this study, we show how proteomic changes in *S. aureus* in response to low-dose pexiganan uncover cellular soft spots that help to identify intervention opportunities. In addition, our findings contribute to the understanding of the early stages of resistance evolution to antimicrobial peptides. Here, we first study the global proteomic response of *S. aureus* to the cationic antimicrobial peptide pexiganan at concentrations similar to and below MIC. This helps us to detect the possible metabolic changes that open the path to collateral sensitivity to nucleoside analogs. We then confirm that these treatments sensitize *S. aureus* to antimetabolite purine and pyrimidines analogs.

## Materials and Methods

### Bacteria and Growth Conditions

We used *S. aureus* SH1000 (26) for all experiments. Bacteria were cultured in non-cation-adjusted (unsupplemented) Mueller–Hinton broth (MHB) as recommended for antimicrobial peptides susceptibility testing (27).

### Global Proteomics by LC-Mass Spectrometry

*Staphylococcus aureus* strain SH1000 was grown in non-cation-adjusted MHB to the mid-exponential-phase (OD_600_ 0.5) at 37°C with vigorous shaking. The cultures were diluted 100 times in fresh MHB in a separate tube to a final volume of 5 ml. Pexiganan was added to tubes for a final concentration of 0.5, 1, 2 and 4 μg/ml (1/8,1/4, 1/2, 1x MIC, respectively) in a final culture volume of 10 ml per tube. Non-treated samples were used as controls. After the addition of pexiganan, all tubes were incubated for 30 min with moderate shaking at 37°C. The pellets were collected by centrifugation at 10,000 × g for 5 min and the supernatant was removed by aspiration using a sterile vacuum line. Fifty microlitre of denaturation urea buffer (6 M urea/2 M thiourea/10 mM HEPES, pH 8.0) were then added to each pellet. The resulting suspensions were transferred to new 1.5 ml Eppendorf tubes and exposed to 5 freeze-thawing cycles alternating between liquid nitrogen and a 37°C water bath. The tubes were centrifuged at 20,000 × g for 10 min and the resulting supernatants were transferred to fresh tubes and used as starting protein material for digestion. Each experimental condition had six independent biological replicates. Approximately 50 μg proteins were processed per sample and were in-solution digested as described elsewhere (28). Denaturation buffer-containing protein solutions were reduced by adding 1 μl of 10 mM DTT (final concentration) and incubated for 30 min. The reactions were then alkylated by adding 1 μl of 55 mM iodoacetamide and incubated for 20 min in the dark. Lysyl endopeptidase (LysC, Wako, Japan) resuspended in 50 mM ABC was added to the digestion reaction in a proportion of 1 μg per 50 μg of total sample protein and incubated for 3 h. The samples were diluted with four volumes of 50 mM ammonium bicarbonate (ABC) and digested overnight with 1 μg of sequencing grade modified trypsin (Promega, USA). All digestion steps were performed at room temperature. The next day, the digestions were stopped by adding final concentrations of 5% acetonitrile and 0.3% trifluoroacetic acid (TFA). The samples were desalted using the Stage-tip protocol as described previously (28), and the eluates were vacuum-dried. Peptides were reconstituted in 10 μl of 0.05% TFA, 2% acetonitrile, and 6.4 μl were analyzed by a reversed-phase capillary nano liquid chromatography system (Ultimate 3000, Thermo Scientific) connected to an Orbitrap Velos mass spectrometer (Thermo Scientific). Samples were injected and concentrated on a trap column (PepMap100 C18, 3 μm, 100 Å, 75 μm i.d. × 2 cm, Thermo Scientific) equilibrated with 0.05% TFA, 2% acetonitrile in water. After switching the trap column inline, LC separations were performed on a capillary column (Acclaim PepMap100 C18, 2 μm, 100 Å, 75 μm i.d. × 25 cm, Thermo Scientific) at an eluent flow rate of 300 nl/min. Mobile phase A contained 0.1% formic acid in water, and mobile phase B contained 0.1% formic acid in acetonitrile. The column was pre-equilibrated with 3% mobile phase B followed by an increase of 3–50% mobile phase B in 50 min. Mass spectra were acquired in a data-dependent mode utilizing a single MS survey scan (m/z 350–1,500) with a resolution of 60,000 in the Orbitrap, and MS/MS scans of the 20 most intense precursor ions in the linear trap quadrupole. The dynamic exclusion time was set to 60 s and automatic gain control was set to 1 × 10^6^ and 5,000 for Orbitrap-MS and LTQ-MS/MS scans, respectively.

MS and MS/MS raw data were analyzed using the MaxQuant software package (version 1.6.4.0) with an implemented Andromeda peptide search engine (29). Data were searched against the reference proteome of *S. aureus* strain NCTC 8352 downloaded from Uniprot (2,889 proteins, taxonomy 93061, last modified September 2017) using label-free quantification and the match between runs option was enabled. Filtering and statistical analysis was carried out using the software Perseus (30). Only proteins with intensity values from at least 3 out of 6 replicates were used for downstream analysis. Missing values were replaced from normal distribution (imputation) using the default settings (width 0.3, down shift 1.8). Student's *T*-tests were performed using permutation-based FDR of 0.05.

### Antimetabolite Nucleosides

In this study, we used four nucleoside analogs. We used the pyrimidine analogs 6-azauracil, gemcitabine, 5-fluorouracil and the purine analog 6-thioguanine. All drugs were purchased from Sigma Aldrich (Germany). 6-azauracil is used as a growth inhibitor of various microorganisms via depletion of intracellular GTP and UTP nucleotide pools (31). Gemcitabine is a chemotherapy medication used to treat different types of cancer. Gemcitabine is a synthetic pyrimidine nucleoside analog in which the hydrogen atoms on the 2′ carbon of deoxycytidine are replaced by fluorine atoms and competitively takes part and disrupts several pathways where pyrimidines are needed (24). 5-Fluorouracil is also used as an anticancer treatment and it works by inhibiting cell metabolism by blocking many pathways, but its major action is the inhibition of the thymidylate synthase. By doing so, the synthesis of the pyrimidine thymidine is stalled, which is an essential nucleoside required for DNA replication (32). 5-Fluorouracil causes a drop on dTMP, causing cells to undergo cell death via thymineless death (32, 33).

### Pexiganan and Antimetabolite Nucleosides Susceptibility Testing

Minimal inhibitory concentration (MIC) was determined by broth micro-dilution method modified for cationic antimicrobial peptides (34). Briefly, 2 μl of the mid-exponential phase culture diluted 1:100 (around 10^5^ bacteria) were inoculated into each well of a polypropylene V-bottom 96-well plates with anti-evaporation ring lids (Greiner Bio-One GmbH, Germany). Prior to inoculation, pexiganan and the analogs (a kind gift from Dr. Michael A. Zasloff, Georgetown University) were 2-fold serially diluted in a final volume of 100 μl MHB per well using 32 μg/ml as starting concentration. Each assay was performed with eight replications and plates were incubated at 37°C in a humid chamber. The MIC was defined as the lowest concentration where no visible bacterial growth was observed after 24 h.

### Isobologram Assay

The combined activity and interactions between peptides, pexiganan, purine and pyrimidine analogs against *S. aureus* in MHB were determined using isobolographic combinations, also called the checkerboard assay method, (8 × 8 matrix of concentrations combinations) (35). In a 96-well plate, 50 μl of pexiganan at 4x MIC concentration was 2-fold serially diluted ranging from 32 to 0.25 μg/ml in the direction of the columns from 1 to 8. In another 96-well plate, 100 μl of nucleoside analogs at 8x MIC concentrations were prepared identically to the previous plate, but diluted in the direction of the rows from A to H. Half of the content (50 μl) of each well from the analog drug plate was transferred to the corresponding well of the plate containing pexiganan in an equal 1:1 mix fashion, halving the concentration of both compounds. In the same plate, the columns 9 and 10 were used to serially dilute both the peptide and the analog drug in the same concentrations that were present in the combination to compare single compounds vs. combination. Columns 11 and 12 were used as a control, by inoculating column 11 wells with bacteria without any drug and leaving columns 12 only with the same volume of MHB as a media contamination control. Each plate was prepared in triplicates to check for consistency. The bacterial suspension was prepared by growing *S. aureus* SH1000 to mid-exponential phase (2.5 h, with moderate shaking at 37°C) in MHB to an OD_600_ between 0.3 and 0.5. The bacterial suspension was diluted in MHB and ~1 × 10^6^ bacteria were inoculated in each well. After 24 h of incubation at 37°C in a humid chamber, the plates were visually examined for growth. The Fractional inhibitory concentration (FIC index) for a combination of pexiganan and each antimetabolite drug was calculated as [(MIC of the peptide in combination with a given analog)/(MIC of peptide alone)] + [(MIC of analog in combination with peptide)/(MIC of analog alone)]. The interpretation of the results was as follow: FIC ≤ 0.5, synergistic; 0.5 < FIC ≤ 1, additive; 1 < FIC ≤ 4, indifferent; FIC > 4, antagonistic (36). To ensure that bacteria lost viability while reading MIC values for pexiganan-analog combinations, we used the resazurin colorimetric assay as described previously with minor modifications (37). Resazurin (THK, Germany) was prepared at 0.015 % in distilled water and sterilized by filtration. It was stored at 4°C for a maximum of 1 week after preparation. Resazurin (0.015%) was added to each well (10 μl per well, 1/3 of the original described quantity) and further incubated for 3 h for the observation of color change. Columns with no color change (blue resazurin) were scored as dead culture. In contrast, color change to purple (reduced resazurin) was considered as a sign of viability.

### Time-Kill Experiments

Starting from early mid-exponential phase cultures (1 × 10^7^ CFU/ml), bacteria were exposed to growing concentrations of pexiganan ranging from 1 to 8x MIC or pexiganan combined with the nucleoside analogs 6-azauracil, gemcitabine, 5-fluorouracil and 6-thioguanine at their respective 1/2x MIC values. The cultures were incubated with soft shaking at 37°C for 2 h. Samples from each culture (1 ml) were taken at 20-min time-point intervals. The samples were diluted and plated to determine cell viability. The experiments consisted of five independent replicates. Non-treated cells were used as a control.

### Statistical Analysis

The effect of treatments on bacterial killing was analyzed using R package nparLD which is designed to perform non-parametric analysis of longitudinal data in factorial experiments modeling the variation over time (38). *P* values of ≤ 0.05, after correction, if needed, were considered statistically significant. All tests were performed with the statistic software R (39).

## Results

### Changes in Protein Profiles After Pexiganan Treatment

We examined *S. aureus* exposed to pexiganan by studying proteome-wide changes after a 30-min treatment with different pexiganan concentrations (0.125, 0.25, 0.5, and 1x MIC, Table S1). Overall, 1160 proteins were identified at a 1% or less false discovery rate (FDR) among which 968 proteins were quantified in at least 3 out of 6 replicates and used for downstream analysis. All identified proteins, their quantification and statistical tests are provided in Table S2. A global overview shows a proteome-wide perturbation induced by pexiganan stress for all concentrations compared to control. Many proteins were significantly differentially expressed (Figure S1). It is noticeable that as long as the dose increases, the level of expression (fold-change) of both overexpressed and suppressed genes, decreases, making the dot scattering of the volcano plot less disperse (Figure S1). This indicates a decrease in the ability of the cell to react with increasing peptide concentration.

### Envelope Stress Response to AMPs

**Figure 1:**
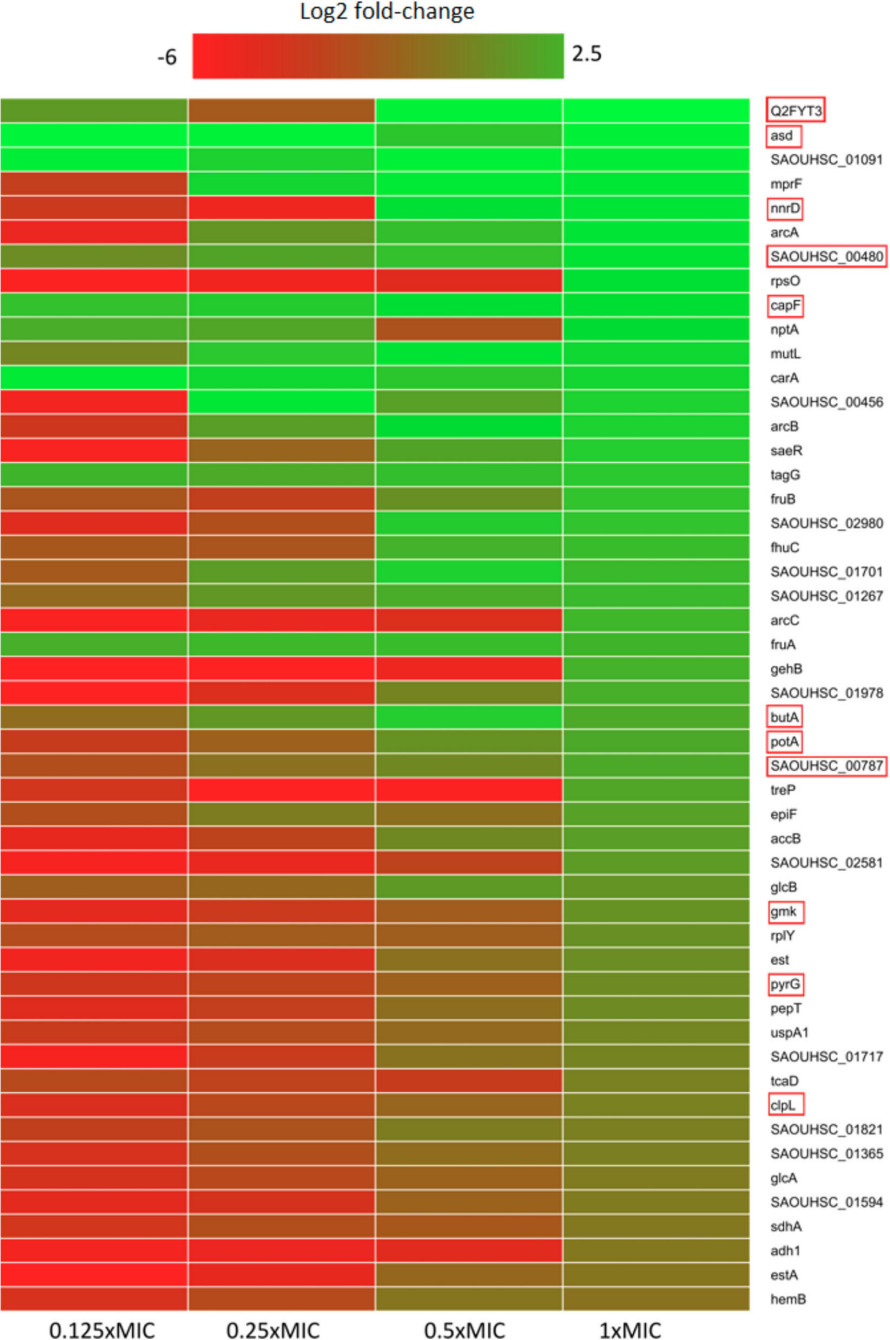
Heatmap of relative protein expression based on label-free quantification detected by liquid chromatography-mass spectrometry (LC-MS). Only the 50 most significantly up-regulated proteins compared to the control are shown (log2 fold-change). Red rectangle highlights proteins that participate in or depend on nucleotide metabolism. Proteins were extracted after 30 min of pexiganan addition (0.125, 0.25, 0.5 and 1 fractions of the minimal inhibitory concentration). log2 fold-changes are given from highest (green) to lowest (red).

Within the upregulated proteome fraction (Figure 1 and Figure S1), a group of proteins related to osmotic stress response shows up. The multi-peptide resistance factor MprF, a protein associated with cationic peptide resistance, which is conserved among many bacterial species (40, 41) is upregulated in all pexiganan doses except in the lowest dose (1/8x MIC). MprF catalyzes the transfer of a lysyl group from L-lysyl-tRNA(Lys) to membrane-bound phosphatidylglycerol producing lysyl-phosphatidylglycerol, a major component of the bacterial membrane with a net positive charge. Hence, a modification of the anionic phosphatidylglycerol with positively charged L-lysine results in the repulsion of the peptides. Changes in the membrane charge is a *per se* resistance mechanism against cationic antimicrobial peptides (42). Thus, MprF increases resistance to moenomycin and vancomycin but also to human defensins (HNP1-3) and contributes to the evasion of oxygen-independent neutrophil killing and other AMPs and antibiotics (43, 44). Another highly expressed protein is CapF, which is involved in the pathway capsule polysaccharide biosynthesis, a mucous layer on the surface of the bacterium that facilitates immune evasion and infection. CapF is an important virulence factor during infections by *S. aureus*. The enzyme CapF is considered a therapeutic candidate to disrupt the capsule polysaccharide biosynthesis (45). Another protein that contributes toward modifying the bacterial envelope and has a significant higher expression is TagG. This protein is part of the wall teichoic acid synthesis during the final steps of the pathway. Wall teichoic acids are important in pathogenesis and play key roles in antimicrobial resistance (41, 46).

### Proteases and Chaperones Proteins

The chaperones/proteases ClpL and TreP are among the fifty upregulated genes for the dose corresponding to the MIC (8 μg/ml). ClpL is an ATP-dependent Clp protease. Clp proteases play a central role in stress survival, virulence and antibiotic resistance of *S. aureus* (47). Another protease induced by pexiganan is PepT, also known as Staphopain A. This enzyme is a cysteine protease that plays an important role in the inhibition of host innate immune response. It cleaves host elastins from connective tissues, pulmonary surfactant protein A in the lungs, and the chemokine receptor CXCR2 on leukocytes (48). Proteolytic cleavage of surfactant protein A impairs bacterial phagocytosis by neutrophils while CXCR2 degradation blocks neutrophil activation and chemotaxis (48, 49). Additionally, PepT promotes vascular leakage by activating the plasma kallikerin/kinin system, resulting in patient hypotension (50).

### Alteration of Metabolism

For example, NptA, a phosphate transporter, usually induced by phosphate limitation, is highly abundant in post-pexiganan treatment. Inorganic phosphate acquisition via NptA is particularly important for the pathogenesis of *S. aureus*. NptA homologs are widely distributed among bacteria and closely related less pathogenic staphylococcal species do not possess this importer. Another phosphate metabolism-related gene with high expression is SAOUHSC_00480, that codes for a putative nucleoside triphosphate pyrophosphohydrolase (51). Also related to phosphate metabolism, we observed a high level of FruA in different pexiganan concentrations. This protein is a phosphoenolpyruvate-dependent sugar phosphotransferase system (a PTS system) is a major carbohydrate active transport system, which catalyzes the phosphorylation of incoming sugar substrates concomitantly with their translocation across the cell membrane and are potentially important for survival in the respiratory tract of the host (52). GlcB, another PTS system is a phosphoenolpyruvate-dependent sugar phosphotransferase system. This protein catalyzes the phosphorylation of incoming sugar substrates and their translocation across the cell membrane (53). Another two phosphate metabolism related proteins, CarA and CarB, which participate in the L-arginine biosynthesis, were induced. They are involved in the first step of the sub-pathway that synthesizes carbamoyl phosphate from bicarbonate. The elevation of these enzymes could indicate that pexiganan stress may be involved in amino acid depletion.

The gene SAOUHSC_00456 that codes for YabA is significantly increased as well by pexiganan. YabA is involved in the initiation of chromosome replication and is a negative controller of DNA replication initiation in *Bacillus subtillis*. YabA and DnaD inhibit helix assembly of the DNA replication initiation protein DnaA (54). The elevated concentration of YabA could stall the cell division while the bacteria is under severe stress. *S. aureus* upregulates Spermidine/putrescine import ATP-binding protein PotA. This protein is part of the ABC transporter complex PotABCD and responsible for energy coupling to the transport system. Spermidine and putrescine are polyamines whose role in *S. aureus* is not well-defined (55). There are also a set of up-regulated proteins coded by the genes SAOUHSC_01717, SAOUHSC_02581, and SAOUHSC_02581 whose functions remain unknown as described in Uniprot database and showed no homology with any known sequence (51).

One of the hallmarks of our proteomic dataset is that we found a higher level of expression, compared to controls, for proteins related with nucleotide metabolism (Figure 1), which is directly connected to the upregulation of phosphate metabolism proteins described above. GmK, for example, is an essential protein for recycling GMP and indirectly, cGMP Guanylate kinase is near four times more abundant than in the control group. GMK is an essential enzyme and a potential antimicrobial drug target owing to its role in supplying DNA and RNA precursors (56). Another nucleobase metabolism-related protein having or exhibiting a higher expression for the 1x MIC treated cells is PyrG. This enzyme catalyzes the ATP-dependent amination of UTP to CTP with either L-glutamine or ammonia as the source of nitrogen. It also regulates intracellular CTP levels through interactions with the four ribonucleotide triphosphates (51).

### Downregulation Response to Pexiganan

Pexiganan also negatively impacted proteome-wide gene expression (Figure S1 and Table S2). Among the most affected gene expressions throughout all concentrations are genes such as *dps* (coding for a known iron storage protein), *hld, copZ, cspC, metQ, sceD, isaA csoB*/*C, dltC*, adsA and *sasG*, SAOUHSCA_01134 and SAOUHSCA_02576. The gene *cspB* codes for the downregulated protein CspD, a cold shock protein that accumulates during low temperature or cold shock. This gene is also a component of the stringent response, indicating that it could be a general stress response gene (57). Other genes showing a differentially low level of expression are SAOUHSC_01986, SAOUHSC_01986, SAOUHSC_008020, SAOUHSC_02093, SAOUHSC_02535, and SAOUHSC_01414 which code for uncharacterized proteins (51). SAOUHSC_01030 is a putative glutaredoxin domain-containing protein but it is not characterized either. The gene SAOUHSC_02576 codes for a putative secretory antigen SsaA, identified in *S. epidermidis* but its function is also unknown (51).

In contrast to the upregulation of peptidoglycan synthesis, we observed that putative peptidoglycan hydrolases and probable lytic transglycosylases IsaA and SceD were downregulated. Interestingly, the *isaA sceD* double mutant is attenuated for virulence, while SceD is essential for nasal colonization in cotton rats (58). The gene *moaD* shows also a reduced level of expression and it codes for a molybdopterin converting factor subunit 1. Molybdopterins are a class of cofactors found in most molybdenum-containing and all tungsten-containing enzymes. Molybdopterin pathway reactions consume guanosine triphosphate that is converted into the cyclic phosphate of pyranopterin (59). Another metabolic enzyme, AldA, aldehyde dehydrogenase central carbohydrate metabolism is downregulated in all doses of pexiganan. This is also the case of CopZ, a chaperone that serves for the intracellular sequestration and transport of copper, delivering it to the copper-exporting P-type ATPase A (CopA) (60).

#### Pexiganan Stress Has a Strong Impact on the Essential Proteome

We visualized the global impact of pexiganan stress (at 1x MIC) on bacterial physiology by using a network analysis based on protein-protein interactions and the function (61) of *S. aureus* essential genes (Figure S3). This network analysis provides global view information on protein level alterations and integrates protein-protein interactions, including indirect functional and direct physical associations (61). At this concentration, it is noticeable that the majority of the essential genes are downregulated, and it is possible that this pattern has a strong influence on pexiganan lethality. The majority of upregulated proteins are ribosomal components.

#### Gene Ontology Analysis Points to an Upregulation of Nucleotide Metabolism

**Figure 2:**
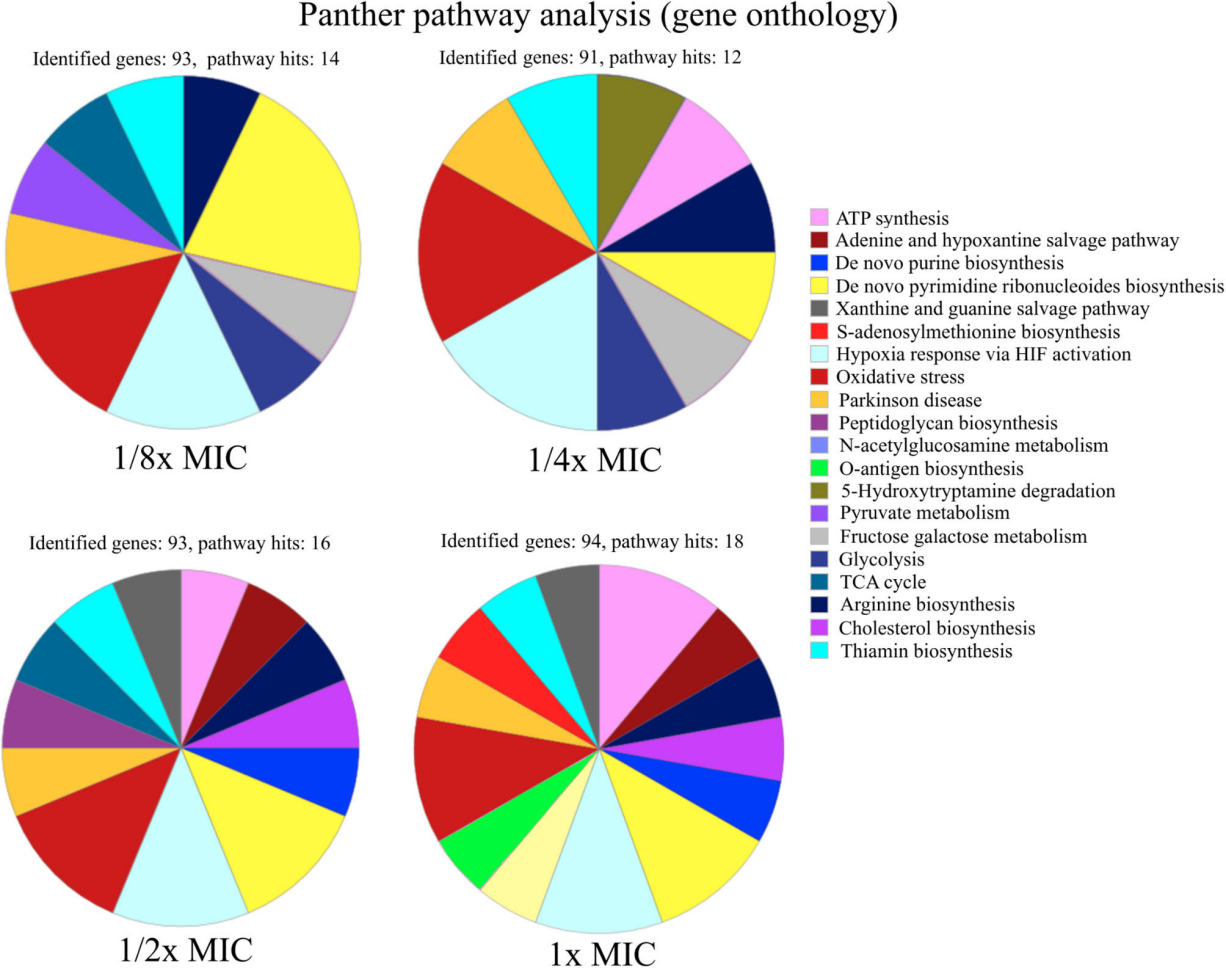
Functional characterization of pathways of up-regulated proteins in *S. aureus* SH1000 at different concentrations of pexiganan (0.125, 0.25, 0.5, and 1 fractions of the minimal inhibitory concentration, MIC). For this analysis, only the 100 most highly differentially expressed proteins for each concentration of pexiganan were used. On top of each chart the number of identified genes and the number of pathway hits (number of genes used for the enrichment analysis) is visible. The analysis was carried out using the online gene ontology analysis software PANTHER (62).

The signature of pexiganan stress on *S. aureus* in the upregulated fraction points to nucleotide metabolism-related genes. GO annotation allows enrichment analysis providing global information based on the gene expression levels by proteomics or transcriptomics or other gene expression datasets (62). We focus this comparative analysis on the protein expression levels of the 100 most upregulated proteins of every pexiganan dosage. We focussed on categorizing by pathways. Some of the upregulated pathways involved genes related to oxidative stress, peptidoglycan synthesis, and N-acetylglucosamine that are expected from cationic antimicrobial peptides since they attack the cell envelopes. In addition, there is a reactivation of the central metabolism by the upregulation of genes from glycolysis, TCA cycle, arginine, and thiamine synthesis. However, the most enriched pathways in the GO analysis for all pexiganan concentrations were related to nucleotide metabolism (Figure 2). The nucleotide upregulated pathways include ATP synthesis, Adenine and hypoxanthine salvage pathways, *de novo* synthesis of purines and pyrimidines and S-adenosylmethionine. This result confirms that pexiganan stress induces a scarcity of these metabolites within the cell. Taking into account the previous results, we hypothesized that upregulation of nucleotide-dependent and related genes could create a collateral sensitivity.

#### Nucleoside Analogs Antimetabolites Act Synergistically With Pexiganan

**Figure 3:**
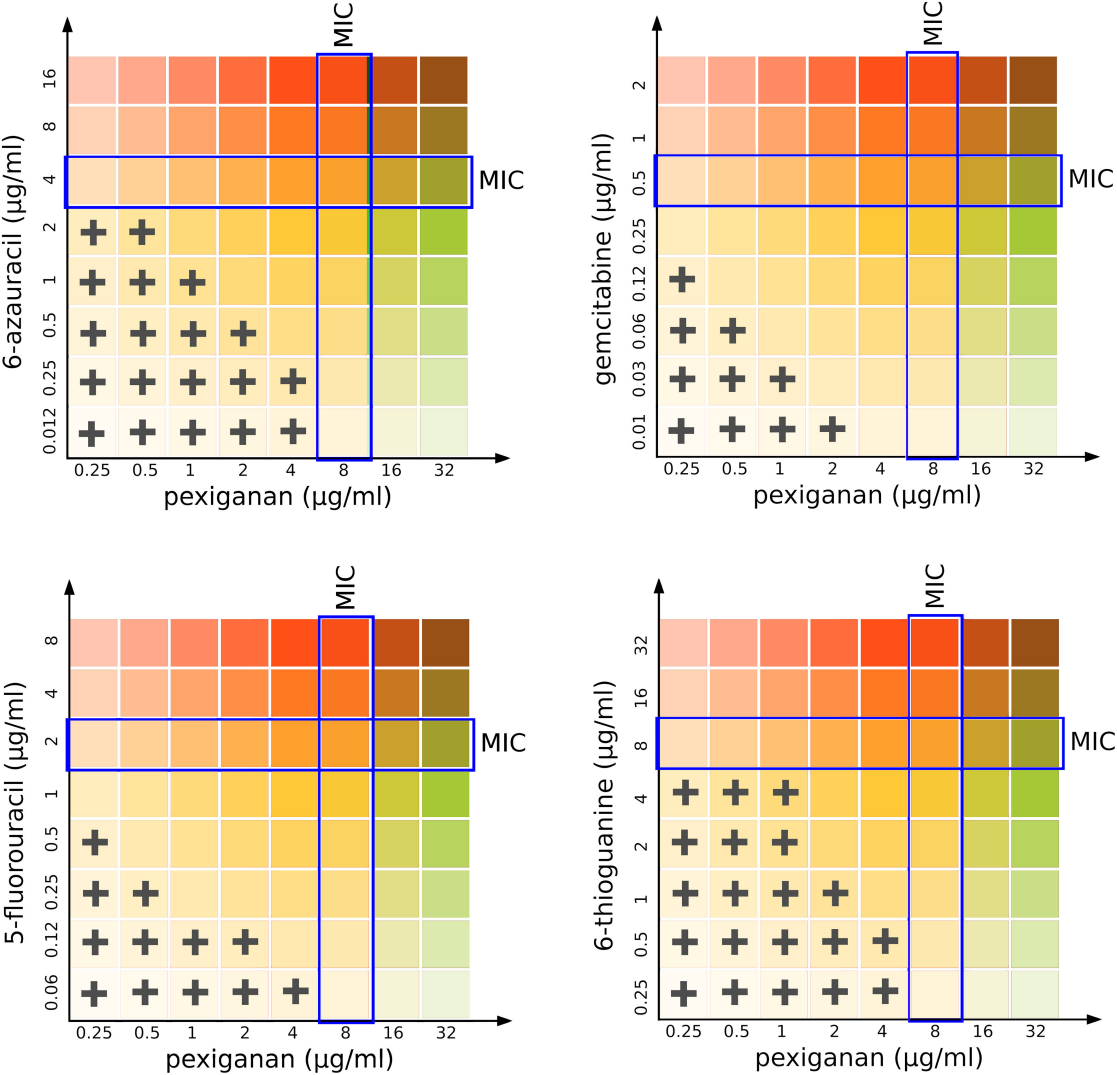
Isobolographical response of pexiganan combination with different antimetabolite nucleosides. The crosses indicate the presence of bacterial growth in the unique concentration combinations of each well. Blue rectangles indicate the MIC value for single drug situations (pexiganan or antimetabolites) and is marked as a reference to visually compare with the actual level of inhibition for each pexiganan-antimetabolite combination.

We designed a simple drug interaction experiment between pexiganan and some nucleoside analogs including the purine and pyrimidine antimetabolites: 6-azauracil, gemcitabine, 5-fluorouracil, and 6-thioguanine (Figure 3, Figure S4 and Table S4). This experiment is the classic isobologram, also known as a checkerboard assay (35). All analogs showed a synergistic activity when combined with pexiganan (Table S4). The most active ones were 5-fluorouracil and gemcitabine and, while the 6-azauracil and 6-thiogunine showed a milder effect according to their respective fractional inhibitory concentration index (Table S4). All the combinations managed to decrease of the minimal inhibitory concentration for each drug when compared to the respective drug alone. These results indicate that pexiganan induces a strong collateral sensitivity to nucleoside analogs.

**Figure 4:**
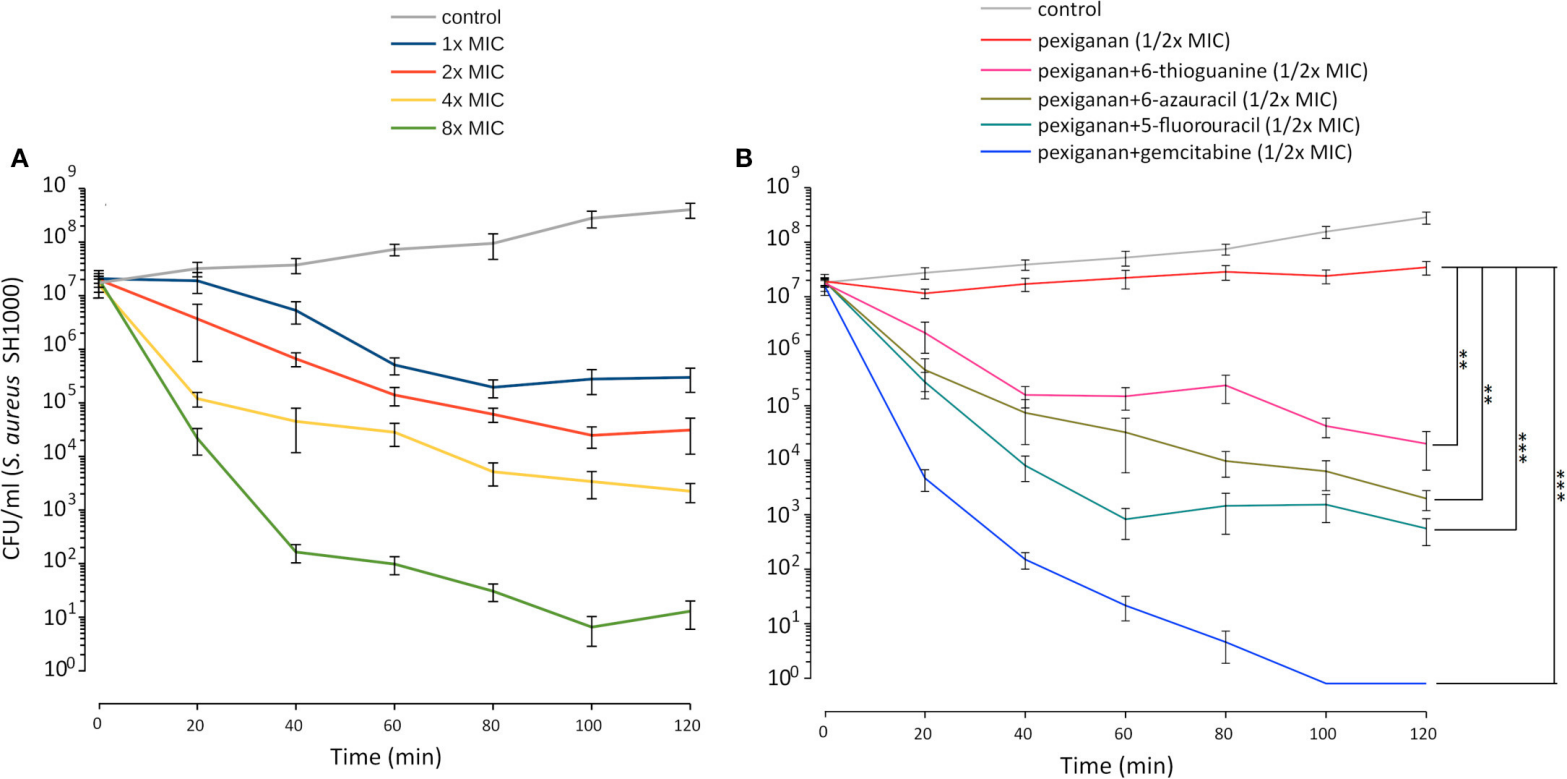
Pexiganan-nucleoside antimetabolite combinations drastically increases the killing capacity of pexiganan. **(A)** Killing dynamic of *S. aureus* SH1000 at different concentrations of pexiganan using the MIC as the starting point. **(B)** Example data of time-kill experiment exposing mid-exponential phase bacterial cultures to pexiganan-nucleoside antimetabolite combinations (both at 1/2x MIC concentrations). The combination has a dramatic effect on the killing ability of pexiganan. Data points were determined by counting colony-forming units (CFU) at different time points. Mean ± SDM, *n* = 5. Asterisks represent significant differences (R package nparLD, one asterisk for *p* < 0.05 and two asterisks for *p* < 0.01 and three asterisks for *p* < 0.001). Only comparisons between pexiganan (1/2x MIC) and pexiganan-analogs combinations are shown.

To study the influence of nucleoside analogs on the killing by pexiganan, we carried out a time-kill experiment combining each of 6-azauracil, gemcitabine, 5-fluorouracil, and 6-thioguanine with pexiganan. We assayed all drugs using half of the minimal inhibitory concentration. We exposed mid-exponential phase *S. aureus* cells to these combinations and sampled the viability of the cultures every 20 min (Figure 4). All compounds significantly increased the killing ability of pexiganan, gemcitabine and 5-fluorouracil being the most active drugs. The killing rate was increased by a few orders of magnitude in all combinations. The killing by the combination of gemcitabine or 5-fluorouracil with pexiganan, at their corresponding half MIC values, was more efficient than 8x MIC concentration of pexiganan alone. The viability was assessed not only by the absence of growth but also by the addition of resazurin, a reagent that turns from blue to purple when it is reduced by microbial enzymes that only work within living bacteria (37).

## Discussion

We have found that pexiganan, a cationic antimicrobial peptide, can induce a stress response in *S. aureus* that results in a proteome-wide impact. Pexiganan treatment upregulates known virulence factors such as MprF, the capsule synthesis protein CapF, a wall teichoic acid TagG, the proteases ClpL and PepT and other proteins important for the interactions with the hosts. This could lead to a phenotypic cross-tolerance of other immune effectors of hosts and possibly complicate the bacterial infection in case of inefficient treatment where bacteria could be exposed to sub-lethal concentrations. This is a legitimate concern since AMP-resistant variants have been reported to have evolved which have shown some cross-resistance with immune system effectors (63, 64). This risk has been shown for pexiganan as well (12). Our data also provides input about possible induced physiological changes that would help *S. aureus* to adapt to the intra-host environment during its interaction with specific immune system effectors.

It is important to note that, given the coverage of the proteomic data and range of pexiganan doses, we did not find evidence of activation of mutagenic stress pathways or recombination. This indicates that the mode of killing by cationic antimicrobial peptide does not increase genome instability as is typical for classic antibiotics (65). We have previously shown and proposed that antimicrobial peptides, including pexiganan, do not increase the rate of either mutagenesis (66) or recombination (67) in Gram-negative bacteria. Our findings here are consistent with these observations in the Gram-positive model bacterium *S. aureus*.

The elevated level of expression of proteins such as GmK, PyrG, NptA and some amino acids-biosynthesis enzymes such as CarA and CarB that participate in the biosynthesis of L-arginine could be explained by changes in permeability. Amino acids, nucleobases and nucleotides are small molecules that could easily escape from the cellular compartment in case of membrane damage. This is a well-known property of cationic agents, including AMPs (68–70). The fact that only a few proteins from the amino acids biosynthesis pathways are upregulated could be explained because the experiments were carried out in a complex medium like MHB that contains several amino acids and bacteria would upregulate only necessary pathways. A similar situation might be expected within a host.

The upregulation of the phosphate and nucleotide-related proteins provides a direction to investigate drug susceptibilities created by pexiganan stress. Although the antimetabolites used in this work have good antibacterial activity, if they are used in monotherapy they are also prone to generate resistance (22, 24). Thus, their use in combination could possibly help to prevent resistance (16, 71).

**Figure 5:**
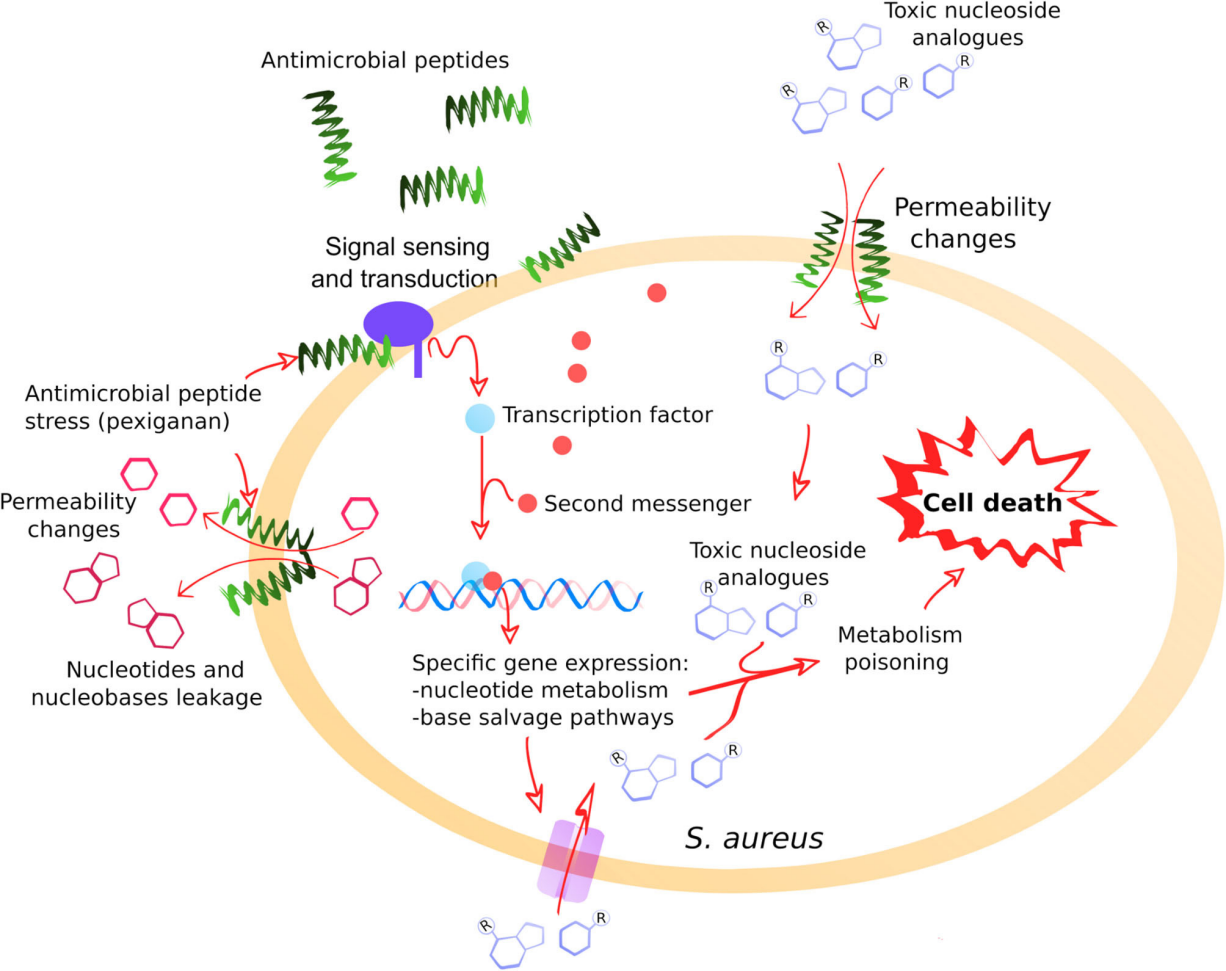
A general model illustrating the positive interaction between pexiganan and nucleoside antimetabolites against *S. aureus*. The interactions of pexiganan with the membrane at sub-inhibitory concentrations lead to transient permeability changes in the envelope that promote leakage of small molecules such as nucleotides, nucleobases or nucleosides. Simultaneously, other small molecules such as toxic nucleoside analogs can increase the diffusion rate toward the intracellular compartment. This stress is sensed by the cell that responds by activating nucleoside metabolism creating an intervention opportunity. In this situation, toxic nucleoside antimetabolites are more efficiently incorporated into RNA, DNA, and other nucleotide depending reactions that may include envelope synthesis, enhancing toxicity and leading to faster cell killing.

The synergistic combined action of pexiganan with nucleoside antimetabolites could be potentially explained by two underlying mechanisms. First, pexiganan stress forces a response by *S. aureus* that upregulates nucleobase salvage pathways and other nucleotide-dependent metabolic pathways. Second, pexiganan has the potential to change membrane permeability and induce the uptake of such metabolites even at sublethal concentrations possibly leading to much higher intracellular concentrations (Figure 5). We have shown previously that cationic antimicrobial peptides can mediate the uptake of small molecules due to changes in permeability at sublethal concentrations (70). The more potent activity of gemcitabine and 5-fluorouracil could be explained because they act on the cell walls as previously reported (24, 72). An additional potential therapeutic advantage of the nucleoside analogs studied here is that all the clinical properties of these drugs are well-known, including toxicological profile, pharmacological activities and metabolizing properties (22, 73). All of them are approved drugs, which should facilitate the introduction of such combinations in clinical practices.

We have shown recently that antimicrobial peptides, including pexiganan, can induce priming in bacteria, an enhanced response to the peptides when bacteria are pre-exposed to low concentrations. We defined the priming response as the ability of bacteria to have better survival to the peptide when it has been exposed to sub-inhibitory concentration in advance. The consequence of priming is not only survival but an increase in tolerance and persistence (74). Tolerance and persistence are a non-genetic path that increase survival to antimicrobials and lead to infection control failure (75). It has been proposed that evolution of tolerance in response to sub-inhibitory concentrations of antibiotics precedes or enhances the emergence of resistance (76). The use of antimetabolites could potentially abolish this property in therapeutic usage.

## Conclusions

The analysis of the pexiganan stress response by *S. aureus* has shown a global response involving several proteins known for their role in the development of resistance against antimicrobial peptides and other immune system effectors. Pexiganan has also shown a synergistic increase of antibacterial activity when it is combined with nucleoside antimetabolites. Taken together, our results suggest that pexiganan renders *S. aureus* susceptible to purine and pyrimidine analogs, which are traditionally used for cancer treatment. These antimetabolites can enhance the bactericidal activity of pexiganan against *S. aureus* under the tested conditions. The significant potentiation of the pexiganan bactericidal activity and the decrease of minimal inhibitory concentrations when compared with pexiganan alone could be the basis for new formulations of pexiganan. These results are probably extendable to other antimicrobial peptides and other bacterial pathogens. Thus, the leakage of nucleotides and intermediate small metabolites or cofactors caused by cationic peptides and nucleotide metabolic pathways are common traits of bacteria-peptide interactions as proposed for the symbiont–host interface (77). Our results also show that understanding how antimicrobials operate and how pathogens respond to them is important to guide the design of new effective therapies. Physiological response by bacteria is informative or suggestive about additional drug combinations that can limit the chances of pathogens to evolve resistance while increasing pathogen clearance and decreasing toxicity. This approach should be exploited to rationally design new antimicrobial combinations.

## Data Availability Statement

The original contributions presented in the study are included in the article/Supplementary Material, further inquiries can be directed to the corresponding author/s.

## Author Contributions

AR-R and JR conceived the study. AR-R, AN, BE, GS, and BK performed the experiments and collected the data. AR-R, GS, JK, BK, and CW analyzed the data. AR-R and JR wrote the manuscript and revised the final document. All authors agree to be held accountable for the content therein and approved the final version.

## Conflict of Interest

The authors declare that the research was conducted in the absence of any commercial or financial relationships that could be construed as a potential conflict of interest.

## Supplementary Material

Figure S1 Volcano plots –log q values vs. log2 fold change of protein intensity measured by LC-MS of pexiganan treated cells with different fractions of the MIC, each compared to an untreated control). Black dots represent not significant expressed proteins while green dots show the upregulated portions and red ones represent the down-regulated fraction.

Figure S2 Heatmap of relative protein expression based on label-free quantification by liquid chromatography/mass spectrometry (LC-MS). Only the 50 most statistically significant down-regulated proteins are shown, taking as a reference the ones from 1xMIC pexiganan concentration (0.125, 0.25, 0.5, and 1 fractions of the minimal inhibitory concentration). Intensity ranges of the log2 fold-changes are given from highest intensity (green) to lowest (red) sorted by their values for the 1x MIC.

Figure S3 Network analysis of pexiganan stress (1x MIC) on essential genes interactome of *S. aureus* SH1000. Pale green nodes indicate upregulated proteins while pale red ones represent down-regulated ones. Gray nodes correspond with genes with a high degree of connectivity with this essential network, but they were not labeled. Note the higher proportion of downregulated genes among essential proteome while the majority of unregulated proteins are ribosomal components and thus they aggregate due to physical interaction. The interaction among nodes shows the proteome-wide impact of pexiganan stress at an inhibitory concentration.

Figure S4 Isobologram showing the synergistic activity of pexiganan and different nucleotide antimetabolite combinations against *S. aureus* SH1000. Columns with no color change (blue resazurin) indicate no viable bacteria while color change to purple (reduced resazurin) was considered as a sign of bacterial growth. Please note that the red rectangle indicates the well of the plates used for the drug combination (8 × 8 wells) while the double arrows indicate the alongside single drug MIC determination. A typical plate is shown from three repetitions. The blue rectangles show the positive (same bacterial inoculum in LB medium) and negative (LB medium alone) controls.
