# Supplementary figures and images for: Antimicrobial Peptide Induced-Stress Renders Staphylococcus aureus Susceptible to Toxic Nucleoside Analogs
Source: Front Immunol. 2020 Sep 29;11:1686. doi: 10.3389/fimmu.2020.01686 (PMC7550632; doi:10.3389/fimmu.2020.01686)

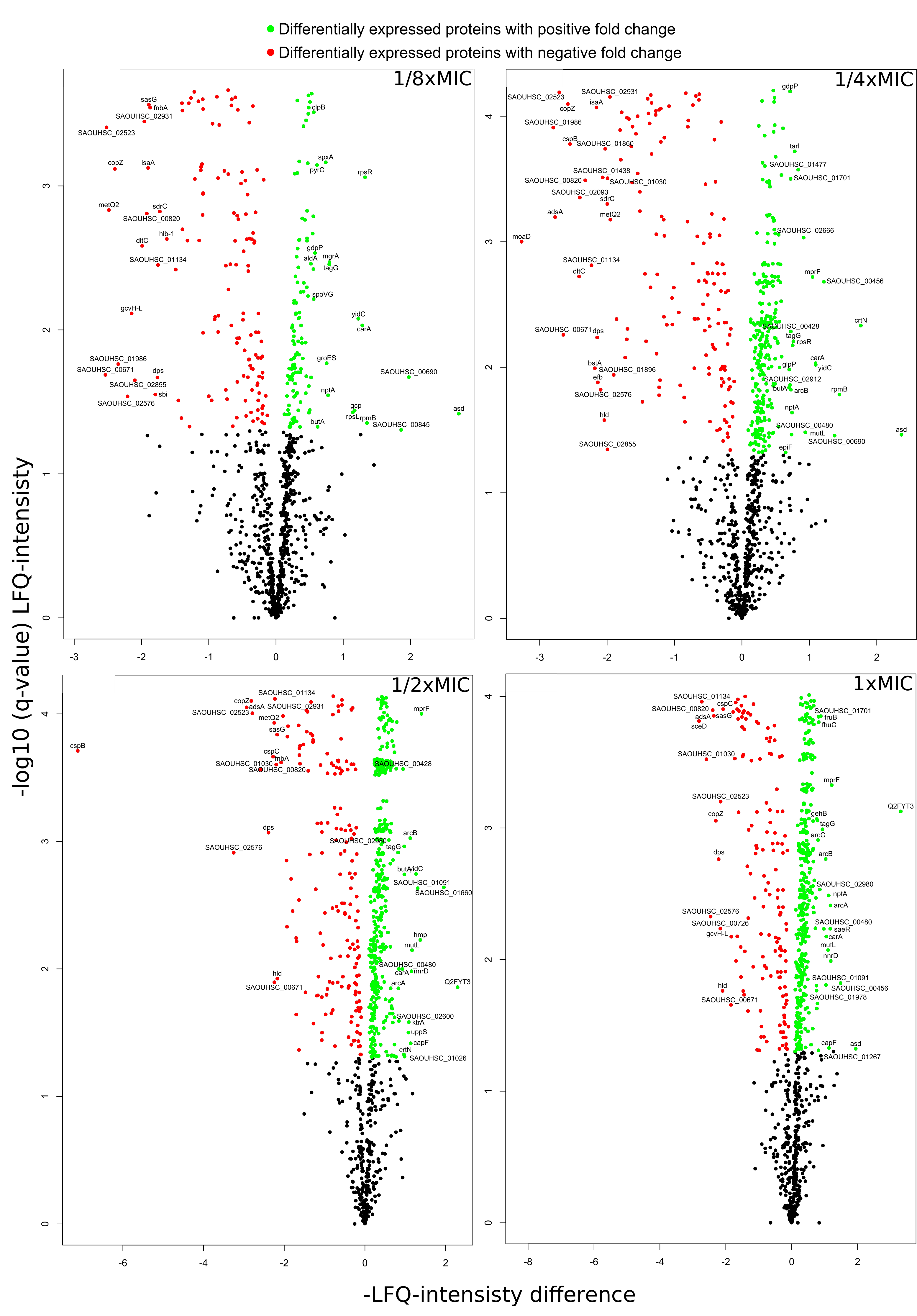

Supplement: Figure S1 — Volcano plots –log q values vs. log2 fold change of protein intensity measured by LC-MS of pexiganan treated cells with different fractions of the MIC, each compared to an untreated control). Black dots represent not significant expressed proteins while green dots show the upregulated portions and red ones represent the down-regulated fraction. [file Image_1.TIFF]

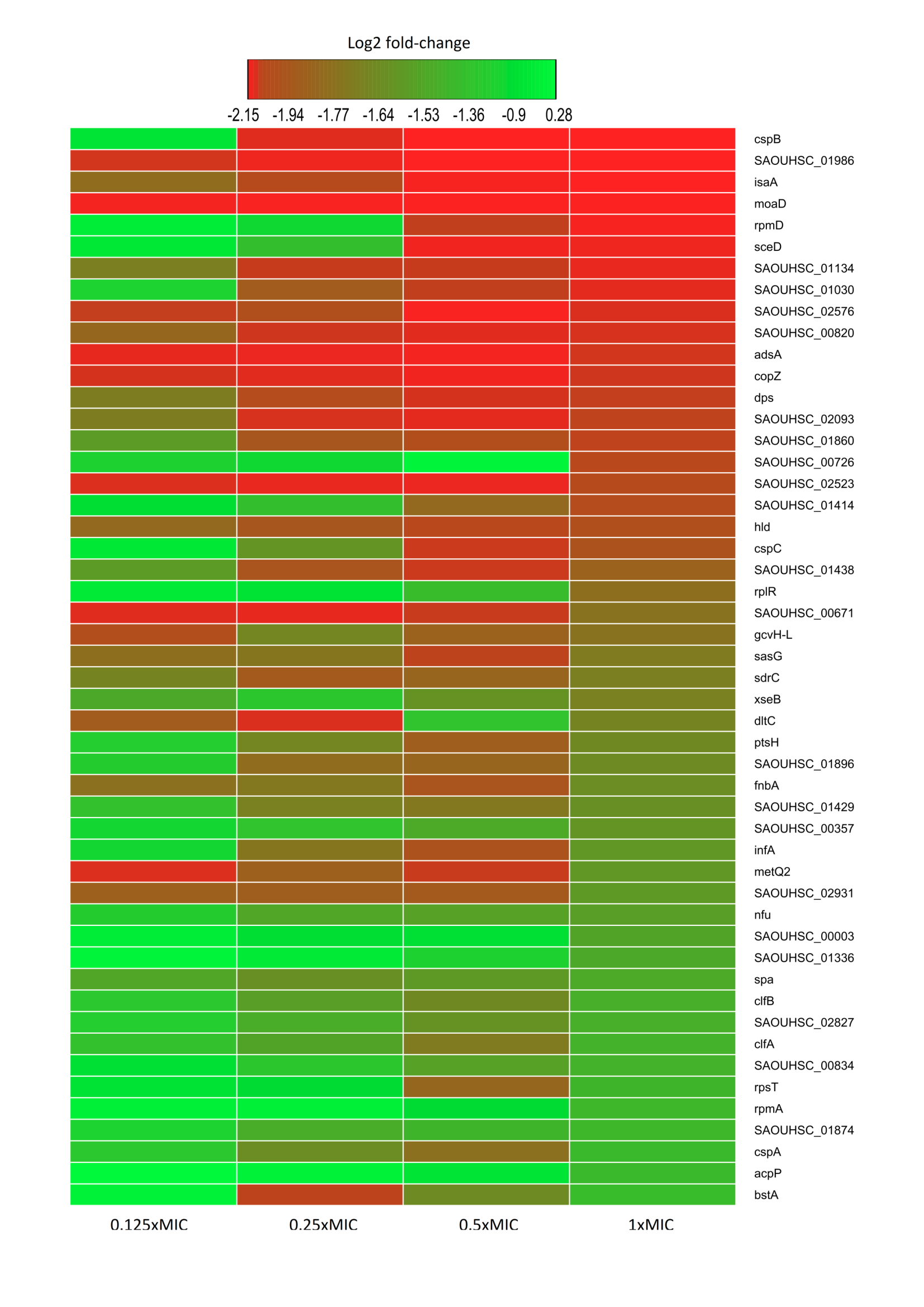

Supplement: Figure S2 — Heatmap of relative protein expression based on label-free quantification by liquid chromatography/mass spectrometry (LC-MS). Only the 50 most statistically significant down-regulated proteins are shown, taking as a reference the ones from 1xMIC pexiganan concentration (0.125, 0.25, 0.5, and 1 fractions of the minimal inhibitory concentration). Intensity ranges of the log2 fold-changes are given from highest intensity (green) to lowest (red) sorted by their values for the 1x MIC. [file Image_2.TIFF]

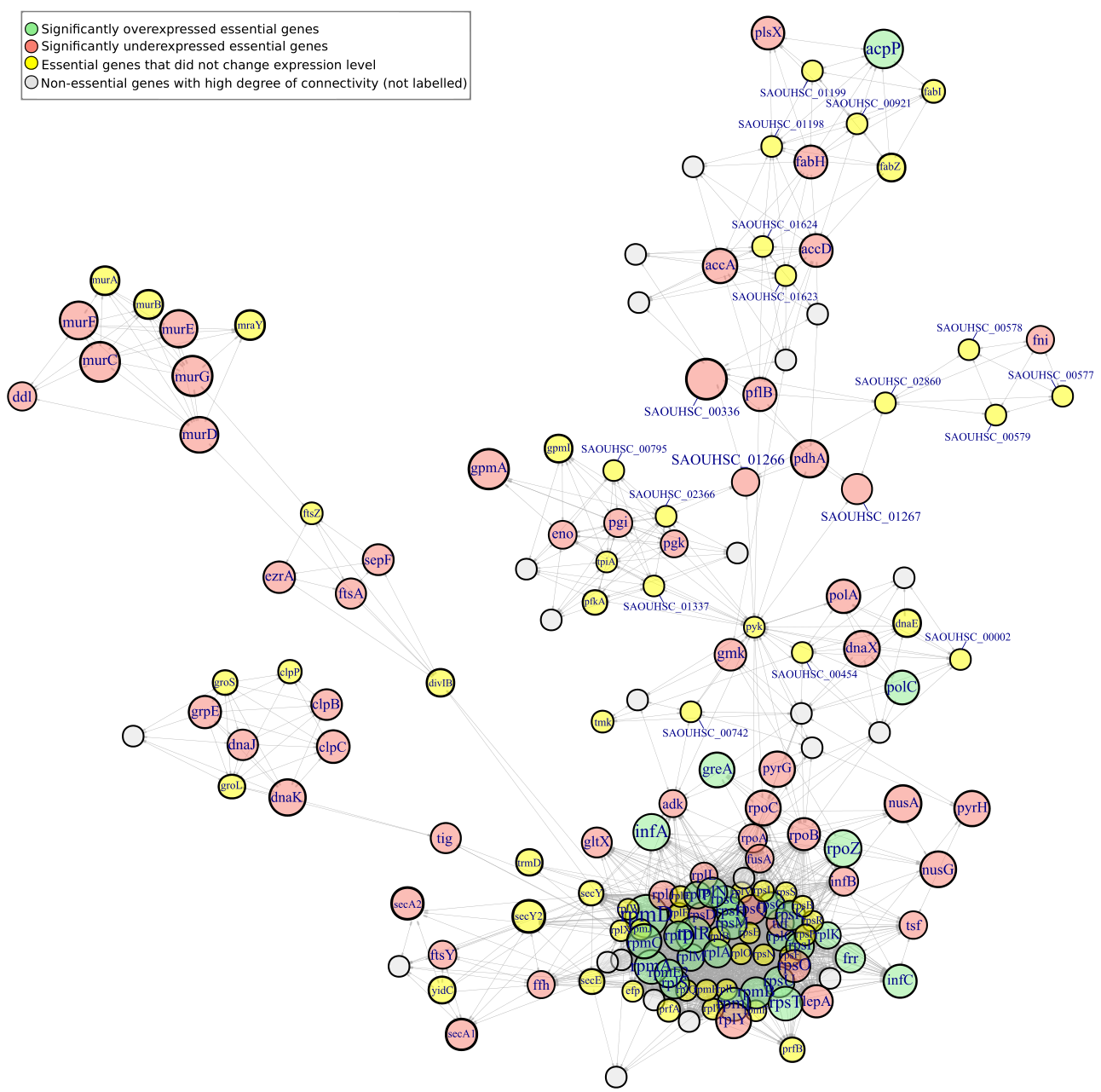

Supplement: Figure S3 — Network analysis of pexiganan stress (1x MIC) on essential genes interactome of S. aureus SH1000. Pale green nodes indicate upregulated proteins while pale red ones represent down-regulated ones. Gray nodes correspond with genes with a high degree of connectivity with this essential network, but they were not labeled. Note the higher proportion of downregulated genes among essential proteome while the majority of unregulated proteins are ribosomal components and thus they aggregate due to physical interaction. The interaction among nodes shows the proteome-wide impact of pexiganan stress at an inhibitory concentration. [file Image_3.TIFF]

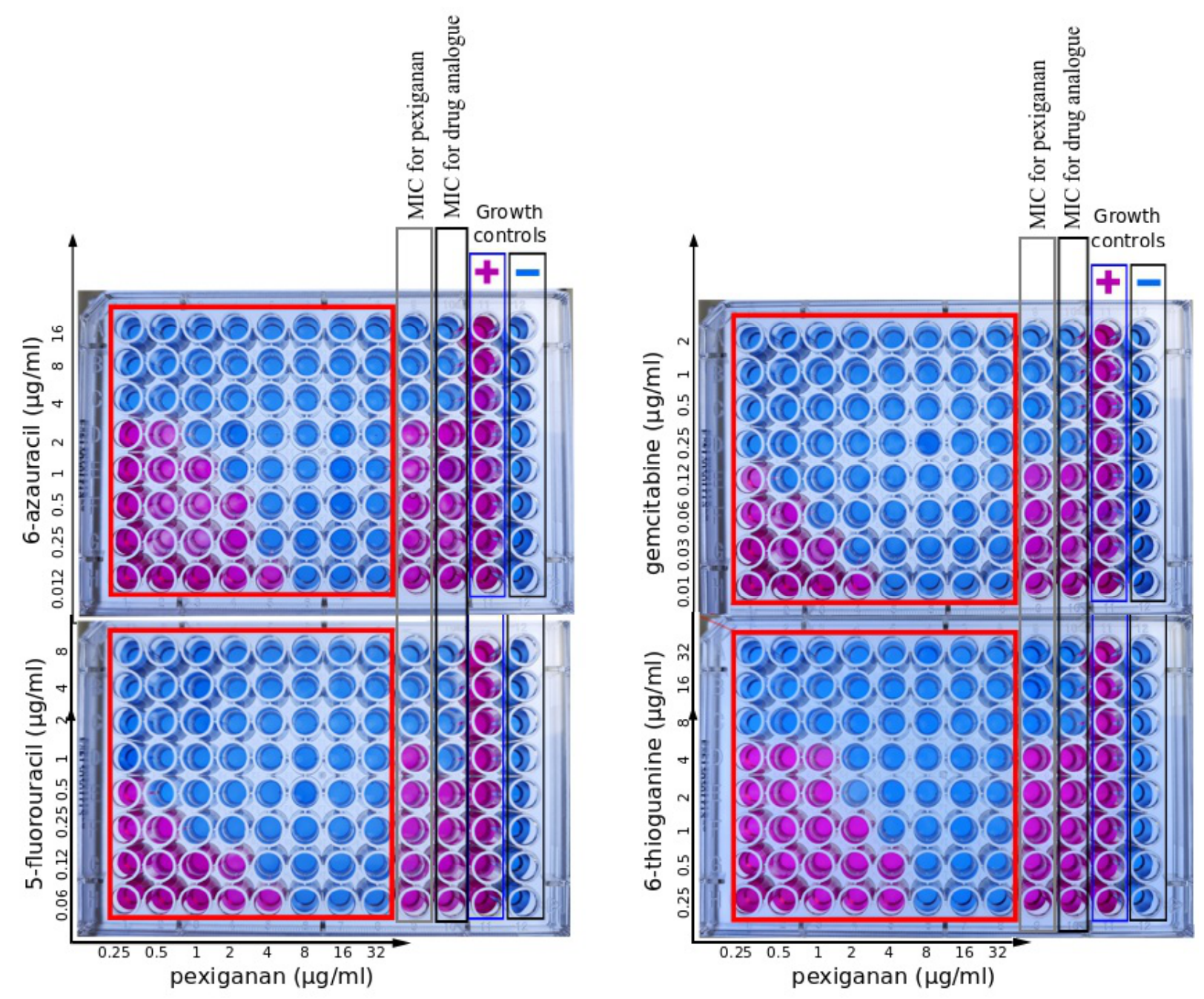

Supplement: Figure S4 — Isobologram showing the synergistic activity of pexiganan and different nucleotide antimetabolite combinations against S. aureus SH1000. Columns with no color change (blue resazurin) indicate no viable bacteria while color change to purple (reduced resazurin) was considered as a sign of bacterial growth. Please note that the red rectangle indicates the well of the plates used for the drug combination (8 × 8 wells) while the double arrows indicate the alongside single drug MIC determination. A typical plate is shown from three repetitions. The blue rectangles show the positive (same bacterial inoculum in LB medium) and negative (LB medium alone) controls. [file Image_4.TIFF]
