# Supplementary material for: Antimicrobial Peptide Induced-Stress Renders Staphylococcus aureus Susceptible to Toxic Nucleoside Analogs
Source: Front Immunol. 2020 Sep 29;11:1686. doi: 10.3389/fimmu.2020.01686 (PMC7550632; doi:10.3389/fimmu.2020.01686)
Supplement: Supplementary file 5 [file Table_1.pdf]

Table S1. Minimum inhibitory concentration (MIC) data for *S. aureus* SH1000 to pexiganan and antimetabolite analogues used in this study.

Drug	MIC (µg/ml)
pexiganan	8
6-azauracil	4
gemcitabine	0.5
5-fluorouracil	2
6-thioguanine	8
