# Supplementary material for: Antimicrobial Peptide Induced-Stress Renders Staphylococcus aureus Susceptible to Toxic Nucleoside Analogs
Source: Front Immunol. 2020 Sep 29;11:1686. doi: 10.3389/fimmu.2020.01686 (PMC7550632; doi:10.3389/fimmu.2020.01686)
Supplement: Supplementary file 7 [file Table_3.pdf]

Table S3. Fractional inhibitory concentration (FIC) data for *S. aureus* SH1000 to pexiganan and antimetabolite analogues used in this study.

**Drug Combination**	**FIC Index**	**Interaction type**
Pexiganan + gemcitabine	0.051	Synergistic
Pexiganan + 6- azauracil	0.25	Synergistic
Pexiganan + 5-fluorouracil	0.037	Synergistic
Pexiganan + 6-thioguanine	0.375	Synergistic
