# Supplementary material for: Antimicrobial Peptide Induced-Stress Renders Staphylococcus aureus Susceptible to Toxic Nucleoside Analogs
Source: Front Immunol. 2020 Sep 29;11:1686. doi: 10.3389/fimmu.2020.01686 (PMC7550632; doi:10.3389/fimmu.2020.01686)
Supplement: Supplementary file 8 [file Table_4.pdf]

Table S4. Antimetabolite analogues used in this study. Properties and doses used in human treatments.

Properties	Antimetabolite analogues			
	**6-azauracil**	**gemcitabine**	**5-fluorouracil**	**6-thioguanine**
**Type**	pyrimidine nucleoside antimetabolite	pyrimidine nucleoside antimetabolite	pyrimidine nucleoside antimetabolite	Analogue of purine base guanine
**Structure**	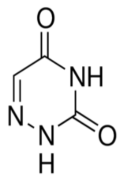	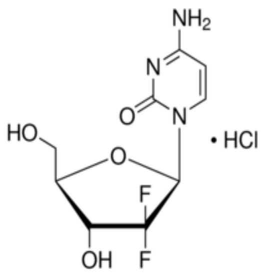	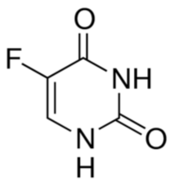	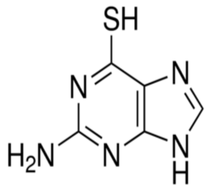
**Chemical formula**	$C_3H_3N_3O_2$	$C_9H_{11}F_2N_3O_4$	$C_4H_3FN_2O_2$	$C_5H_5N_5S$
**Uses**	Antitumor activity against sarcoma, lymphoma, adenocarcinoma	Broad spectrum antitumor activity and a low toxicity profile	Widely used in the treatment of cancers such as colorectal, breast and aerodigestive tract.	Used for Acute myelogenous leukemia and may be used in chronic myelogenous leukemia**.**
**Mode of action**	Leads to decreased intracellular GTP levels	killing cancer cells during DNA synthesis, inhibits ribonucleotide reductase	inhibiting essential biosynthetic processes by mimicking uracil and thymidine and affect DNA and RNA function	Mimicking of guanine and upon DNA incorporation results in damage inducing replication failure.
**Doses**	18 mg/kg to 1268 mg/kg	Given as IV: 27.027 mg/kg	Given as IV: 5.4-16.21 mg/kg	Tablets 2mg/kg
**References**	(Shnider et al., 1960)	(Toschi et al., 2005)	(Longley et al., 2003)	(Munshi et al., 2014)

References

Longley, D. B., Harkin, D. P., & Johnston, P. G. (2003). 5-Fluorouracil: Mechanisms of action and clinical strategies. In *Nature Reviews Cancer* (Vol. 3, Issue 5, pp. 330–338). Nat Rev Cancer. https://doi.org/10.1038/nrc1074

Munshi, P. N., Lubin, M., & Bertino, J. R. (2014). 6-Thioguanine: A Drug With Unrealized Potential for Cancer Therapy. *The Oncologist*, *19*(7), 760–765. https://doi.org/10.1634/theoncologist.2014-0178

Shnider, B. I., Frei Iii, E., Tuohy, J. H., Gorman, J., Freireich, E. J., Brindley, C. O., & Clements, A. (1960). *Clinical Studies of 6-Azauracil*. Cancer Research. 1960. 20, 28-33.

Toschi, L., Finocchiaro, G., Bartolini, S., Gioia, V., & Cappuzzo, F. (2005). Role of gemcitabine in cancer therapy. *Future Oncology*, *1*(1), 7–17. https://doi.org/10.1517/14796694.1.1.7
